# Enhancing vertebral fracture prediction using multitask deep learning computed tomography imaging of bone and muscle

**DOI:** 10.1007/s00330-025-12049-3

**Published:** 2025-12-01

**Authors:** Sung Hye Kong, Saemee Choi, Wonwoo Cho, Sung Bae Park, Seung Shin Park, Jaegul Choo, Jung Hee Kim, Sang Wan Kim, Chan Soo Shin

**Affiliations:** 1 Department of Internal Medicine, Seoul National University Bundang Hospital, Bundang, Gyeonggi-do Republic of Korea; 2 Department of Internal Medicine, Seoul National University College of Medicine, Seoul, Republic of Korea; 3 Kim Jaechul Graduate School of AI, Korea Advanced Institute of Science and Technology, Daejeon, Republic of Korea; 4 Letsur Inc., Seoul, Republic of Korea; 5 Department of Medical Device Development, Seoul National University College of Medicine, Seoul, Republic of Korea; 6 Department of Neurosurgery, Seoul National University Boramae Hospital, Seoul, Republic of Korea; 7 Department of Internal Medicine, Seoul National University Hospital, Seoul, Republic of Korea; 8 Department of Internal Medicine, Seoul National University Boramae Hospital, Seoul, Republic of Korea

**Keywords:** Fracture, Prediction, Deep learning, Longitudinal cohort, Fracture risk assessment

## Abstract

**Objectives:**

To develop and externally validate a computed tomography (CT)-based multitask learning model to predict fracture risk.

**Materials and methods:**

This study was conducted in two parts, using a multitasking learning approach. We developed a cross-sectional vertebral fracture (VF) detection model using abdominal CT scans of 2553 patients aged 50–80 years. Then, we leveraged this detection model within a multitask learning framework to develop a longitudinal VF prediction model over a 5-year follow-up period. External testing was performed on 1506 patients from two independent hospitals. The performance was compared between the single-task and multitask models, bone-only and bone+muscle images, and image-only and clinical models.

**Results:**

For the cross-sectional fracture detection model, the mean age of the patients was 76.2 years, and 66.7% were female. In the classification task for detection of VF, the model using both bone and muscle showed an area under the receiver operating characteristic curve (AUROC) of 0.82 in the development set and 0.80 in the external test sets. Using multitask learning, the bone + muscle image model showed a c-index of 0.68 and had superior performance than the bone-only model in the external test set for 2-year, 3-year, and 5-year AUROCs (0.79 vs. 0.75, 0.71 vs. 0.68, and 0.71 vs. 0.68, respectively, all *p* < 0.01). Also, the multitask model significantly outperformed the Fracture Risk Assessment Tool (FRAX) (c-index: 0.68 vs. 0.66, *p* < 0.01).

**Conclusion:**

The CT-based multitask learning model integrating both bone and muscle data showed superior predictive performance for VFs compared with models using bone images only and traditional clinical models.

**Key Points:**

***Question*** *Vertebral fracture risk remains underestimated in many individuals undergoing CT scans for other reasons, highlighting the need for improved opportunistic prediction tools.*

***Findings*** *A multitask deep learning model integrating both bone and muscle features from CT scans demonstrated superior performance compared to bone-only and traditional clinical models, including FRAX.*

***Clinical relevance*** *The proposed model enables accurate vertebral fracture risk prediction using routinely acquired CT scans, facilitating early identification and intervention without the need for additional tests.*

**Graphical Abstract:**

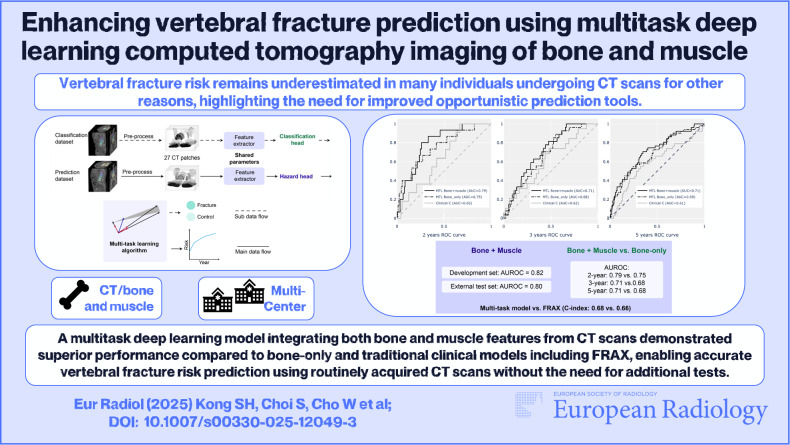

## Introduction

With the aging global population, fragility fractures have increased, substantially impacting healthcare systems, communities, and patient families [1–3]. Therefore, the proactive identification of high-risk individuals is essential for effective prevention strategies and better health outcomes. Although dual-energy X-ray absorptiometry (DXA) is the gold standard for diagnosing osteoporosis [4], many patients do not undergo a DXA scan; alarmingly, 60% of those with major osteoporotic fractures (MOFs) do not receive adequate treatment or interventions to reduce their risk [5].

Opportunistic CT scans are a promising method for identifying patients at high risk of fractures, especially as their use increases with the aging global population [6]. Extracting fracture risk information from these scans is cost-effective because it requires no additional resources and can be performed retrospectively. This approach could streamline the identification of high-risk patients and complement conventional measures such as bone mineral density (BMD) [7]. While several studies have used opportunistic CT to assess BMD by analyzing the attenuation data of the spinal trabecular bone [8, 9], these scans have the additional advantage of assessing the surrounding structures, such as muscles. Recent studies have highlighted that muscles, in addition to bones, play a crucial role in fracture risk, underscoring the importance of including muscle imaging in assessments [10]. On the other hand, the Fracture Risk Assessment Tool (FRAX), developed by the World Health Organization, is widely used in clinical practice to estimate 10-year probabilities of hip and MOFs based on clinical risk factors, with or without BMD input [11]. However, FRAX may not fully capture imaging-based structural and compositional features, such as trabecular degradation or muscle quality, which are increasingly recognized as important determinants of fracture risk.

Building on these advantages, our study aimed to develop and externally test a CT-based model using multitask learning to detect fractures and predict future fracture risk simultaneously. We aimed to enhance the identification of high-risk patients undergoing opportunistic CT scans by incorporating bone and muscle data within a multitasking learning framework. This approach leverages the shared information between tasks to improve model performance, ultimately providing a comprehensive tool for fracture risk assessment.

## Materials and methods

### Study design and participants

**Fig. 1:**
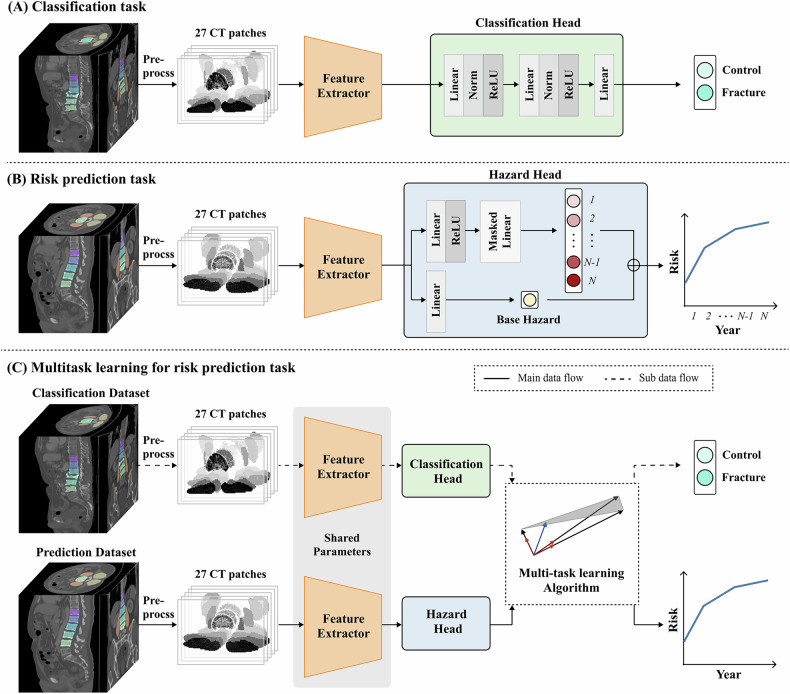
Architecture of (**A**) classification, (**B**) risk stratification, and (**C**) multi-task learning models for vertebral fracture prediction. CT, computed tomography

The study was mainly composed of two parts: a cross-sectional analysis focused on fracture detection and a longitudinal analysis aimed at fracture prediction. The features extracted during the cross-sectional fracture detection analysis were combined with additional information from a longitudinal dataset for use in fracture prediction analysis (Fig. 1). Each task was externally validated using data from two independent hospitals.

First, this cross-sectional analysis was based on a retrospective longitudinal cohort of 32,435 patients who underwent abdominal CT imaging at the Seoul National University Bundang Hospital (SNUBH) between 2010 and 2019. Patients who met all the inclusion criteria were included in the study. The inclusion criteria were as follows: (1) patients who had undergone abdominal CT imaging at SNUBH between 2010 and 2019, and (2) those aged between 50 and 80 years. Patients were excluded if they met any of the following criteria: (1) age < 50 or > 80 years (*n* = 2643), (2) follow-up period of less than 1 year (*n* = 8029), (3) history of spinal surgery at baseline (*n* = 1208), or (4) poor image quality (*n* = 906). Among them, 1144 had baseline compression fractures. Using the propensity score method, an age-, sex-, and body mass index (BMI)-matched control group (*n* = 1409) without compression fractures was included as the development set in the first part of the study. Additionally, an external test set was used to validate the findings, comprising 1506 patients from the Seoul National University Boramae Hospital (*n* = 393) and Seoul National University Hospital (*n* = 1113), using the same protocol between 2015 and 2017 (Supplementary Fig. 1A).

In the second part of the study, a longitudinal analysis focusing on fracture prediction was conducted. Among the 18,505 patients who did not experience fractures at baseline, 693 experienced vertebral fractures (VFs) during the follow-up period, whereas 17,812 did not. After excluding 121 patients owing to poor image quality or inappropriate CT protocols, 572 patients remained in the fracture group. For the control group, individuals were selected from the same time frame as those in the fracture cases. Among the 17,812 patients who did not experience fractures, 1952 age-, sex-, and BMI-matched individuals with the same follow-up period were selected. Fracture events were determined by reviewing medical records, with efforts made to exclude trauma-associated fractures. If the patients underwent multiple CT scans during the follow-up period, the earliest CT scan was performed. Additionally, an external test set of 759 patients from Seoul National University Boramae Hospital (*n* = 550) and Seoul National University Hospital (*n* = 209) was used, following the same protocol between 2015 and 2017 (Supplementary Fig. 1B).

The study protocol was approved by the Institutional Review Board of Seoul National University Bundang Hospital (IRB No. B-2104-677-402). The requirement for informed consent was waived owing to the retrospective nature of the study. This study was conducted in accordance with the ethical standards outlined in the Declaration of Helsinki and the Ethical Principles for Medical Research. This study also adhered to the CLAIM (Checklist for Artificial Intelligence in Medical Imaging) guidelines to ensure transparency and reproducibility of the AI methodology.

### Primary outcome and clinical factors

The primary outcome of this study was the predictive accuracy of VF events occurring between T12 and L4 within five years. Vertebral fractures were defined as morphometric fractures confirmed using radiography or CT. These images were assessed by SHK, SBP, SSP, and JHK, who were blind to the patient information prior to the evaluation. Sociodemographic factors, BMI, social history, and underlying diseases were obtained by reviewing electronic medical records at baseline. Definitions of morphometric VFs and clinical factors are detailed in the Supplementary Material. Measurements of BMD, CT, and calculation of FRAX are described in the Supplementary Material.

### Deep learning techniques and preprocessing of the images

We propose a deep learning-based framework for VF risk prediction that leverages a multitask learning approach to jointly optimize VF detection and fracture risk prediction (Fig. 1). For better understanding, we first outline single-task learning (STL), where the model is trained solely on the fracture risk prediction dataset using a traditional supervised learning setup. In contrast, our multitask learning framework combines datasets for both tasks, enabling the model to extract shared and generalizable features across them. These shared features were then processed by a hazard head to estimate the cumulative probability of VFs within a five-year period. To achieve this, we constructed labeled 3D CT datasets for both tasks and implemented a preprocessing pipeline to facilitate efficient feature extraction. The preprocessed images were passed through a convolutional neural network (CNN)-based feature extractor that outputs shared features. These features are routed to either a classification head for fracture detection or a hazard prediction head for survival analysis, depending on the task. By optimizing both tasks simultaneously, the model learned to predict fracture presence and fracture risk more effectively. We employed the ConvNeXT Tiny architecture (28.6 M parameters, pretrained on ImageNet-1K) as the feature extractor, given its state-of-the-art performance in medical imaging tasks. To ensure robust evaluation, each experiment was repeated 10 times with different random seeds, and the average performance was reported. The image preprocessing and implementation are detailed in the Supplementary Material section.

### Statistical analyses

The variables between groups were compared using the Student’s *t*-test. For fracture detection, the performance of the deep learning model was evaluated using the area under the receiver operating characteristic (ROC) curve, as well as accuracy, sensitivity, and specificity. To determine the cutoff value for computing accuracy, sensitivity, and specificity, we selected the threshold that maximized the difference between the true positive rate and the false positive rate. For the VF risk prediction task, model performance was evaluated using the concordance index (*c*-index), 2-year area under the receiver operator curve (AUROC), 3-year AUROC, and 5-year AUROC. Thresholds for classification metrics were determined using Youden’s J statistic. Statistical analyses were performed using PyTorch, Scikit-learn, and Lifeline libraries in Python. The statistical analyses are described in detail in the Supplementary Material.

## Results

### Clinical characteristics of development and test sets

For the classification task, the development set comprised 2553 patients, whereas the external test set included 1506 patients (Supplementary Table 1). As the patients were age-, sex-, and BMI-matched, age, sex, and BMI were similar between the patients with and without baseline fractures. However, patients in the external test set were significantly younger than those in the development set (74.3 ± 9.2 vs. 77.4 ± 9.6, *p* < 0.01). BMD was consistently lower in patients with fractures across both datasets for the lumbar spine, femoral neck, and total hip (all *p* < 0.01).

For the prediction task, Table 1 outlines the clinical characteristics according to the incident fracture status during follow-up. Patients with incident fractures in the development set had a higher prevalence of current smoking (28.3% vs. 21.6%, *p* = 0.01) and steroid use (17.1% vs. 6.1%, *p* < 0.01). In the external test set, significant differences were noted in steroid use (32.5% vs. 21.0%, *p* = 0.03) and BMI (22.6 ± 3.0 vs. 23.8 ± 3.6 kg/m², *p* = 0.01). Additionally, FRAX scores for MOFs and hip fractures were significantly higher in patients with incident fractures than in those without, only in the development set (development set, FRAX MOF: 7.0 ± 4.4 vs. 6.0 ± 3.8, *p* < 0.01; FRAX hip: 3.1 ± 3.0 vs. 2.6 ± 2.4, *p  *= 0.01).

**Table 1:** Clinical characteristics according to incident fracture for the prediction task

	Development set				External test set				
	Incident fracture (−)	Incident fracture (+)	Total	*p*^*a*^	Incident fracture (−)	Incident fracture (+)	Total	*p*^*a*^	*p*^*b*^
Number of patients	1952	572	2524		682	77	759		
Age	71.7 ± 8.9	72.2 ± 8.5	71.8 ± 8.9	0.30	65.1 ± 9.8	66.3 ± 8.7	65.2 ± 9.7	0.28	0.01
Female	1274 (65.3%)	369 (64.5%)	1643 (65.1%)	0.78	510 (74.8%)	56 (72.7%)	566 (74.6%)	0.80	0.01
Height, cm	158.1 ± 8.7	158.0 ± 8.2	158.1 ± 8.6	0.67	156.7 ± 8.1	157.3 ± 7.2	156.8 ± 8.0	0.59	0.01
Weight, kg	58.9 ± 10.3	58.4 ± 10.0	58.8 ± 10.2	0.31	58.7 ± 10.2	55.9 ± 8.8	58.4 ± 10.1	0.02	0.29
BMI, kg/m^2^	23.5 ± 3.4	23.4 ± 3.5	23.5 ± 3.5	0.46	23.8 ± 3.6	22.6 ± 3.0	23.7 ± 3.6	0.01	0.22
Current smoker	422 (21.6%)	162 (28.3%)	584 (23.1%)	0.01	43 (6.3%)	7 (9.1%)	50 (6.6%)	0.49	< 0.01
Current drinker	417 (21.4%)	124 (21.7%)	541 (21.4%)	0.92	76 (11.2%)	11 (14.3%)	87 (11.5%)	0.53	< 0.01
Use of steroids	119 (6.1%)	98 (17.1%)	217 (8.6%)	< 0.01	143 (21.0%)	25 (32.5%)	168 (22.1%)	0.03	< 0.01
Secondary osteoporosis	138 (7.1%)	69 (12.1%)	207 (8.2%)	< 0.01	27 (4.3%)	2 (3.4%)	29 (4.2%)	0.99	< 0.01
Follow-up duration, yrs	3.7 ± 2.1	3.8 ± 2.2	3.7 ± 2.1	0.32	4.7 ± 2.5	4.4 ± 2.5	4.6 ± 2.5	0.28	< 0.01
FRAX, MOF	6.0 ± 3.8	7.0 ± 4.4	6.3 ± 4.0	< 0.01	4.8 ± 3.6	5.5 ± 4.9	4.9 ± 3.7	0.47	< 0.01
FRAX, hip	2.6 ± 2.4	3.1 ± 3.0	2.7 ± 2.6	0.01	4.6 ± 3.3	4.3 ± 2.5	4.6 ± 3.3	0.70	< 0.01

Incident fracture (−) and (+) groups represent participants who did not and did experience fractures at 5 years of follow-up, respectively. Use of steroids was defined as the use of prednisolone 5 mg daily or equivalent over 3 months. Numbers are presented as numbers (percentages) or mean (standard deviation). The variables between groups were compared using the Student *t*-test for continuous variables and the *χ*^2^ test for categorical variables. *p*^*a*^ represents the *p*-value between groups with and without baseline fractures. *p*^*b*^ represents the *p*-value between the development and external test sets

*BMI* body mass index, *FRAX* fracture risk assessment, *MOF* major osteoporotic fracture

### Comparison of bone-only and bone + muscle model performance for baseline fracture detection

The performance of the image-based models in detecting baseline fractures is presented in Supplementary Table 2. In the development set, the Bone + Muscle model showed a superior AUROC (0.82 ± 0.01) compared to the Bone-only model (0.81 ± 0.01, *p* = 0.01). Similar trends were observed in the external test set, with the Bone + Muscle model demonstrating a higher AUROC (0.80 ± 0.01 vs. 0.76 ± 0.01, *p* < 0.01). In addition, the Bone + Muscle model exhibited higher specificity than the Bone-only model in the development set (0.83 ± 0.01 vs. 0.66 ± 0.01, *p* < 0.01). In the test set, the Bone + Muscle model outperformed the Bone-only model in both sensitivity (0.60 ± 0.01 vs. 0.68 ± 0.01, *p* < 0.01) and specificity (0.84 ± 0.01 vs. 0.71 ± 0.01, *p* < 0.01).

### Comparison of single-task and multitask model performance for vertebral fracture prediction

**Fig. 2:**
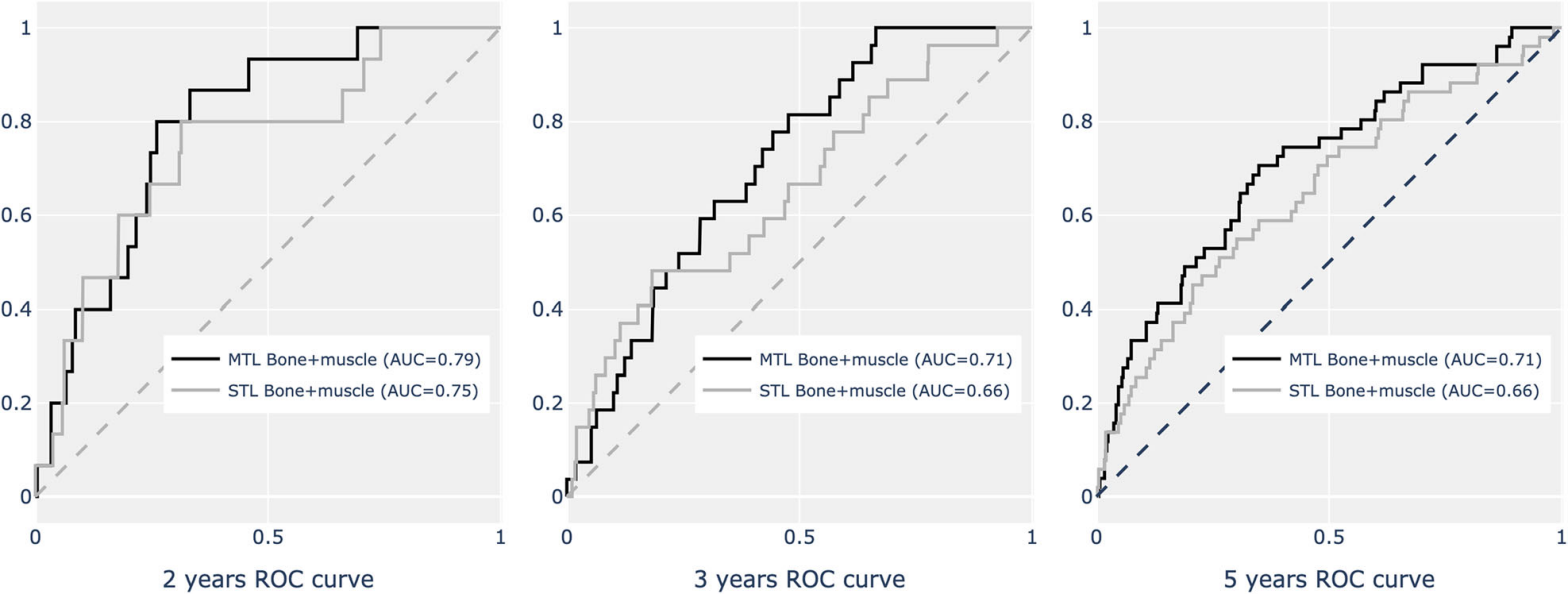
Receiver operating characteristic curves comparing single-task and multi-task machine learning models for predicting vertebral fracture. MTL, multi-task learning; STL, single-task learning

Next, we compared the performance of image-based models in predicting VFs in both the development and external test sets, focusing on single- and multitask learning approaches (Table 2 and Fig. 2). In the STL models, the bone-only and bone + muscle models performed similarly in terms of the *c*-index for both the development (0.69 vs. 0.70, *p* = 0.40) and external test sets (0.65 vs. 0.65, *p* = 0.40). In addition, in the external test set, the bone + muscle model outperformed the bone-only model for the 2-year AUROC (0.72 vs. 0.76, *p* < 0.01).

**Table 2:** Comparisons of the performances of image models in predicting vertebral fractures

	Development set			External test set		
	Bone-only	Bone + Muscle	*p*	Bone-only	Bone + Muscle	*p*
Single-task learning						
* c*-index	0.69 ± 0.02	0.7 ± 0.02	0.40	0.65 ± 0.02	0.65 ± 0.01	0.40
2y-AUROC	0.72 ± 0.03	0.73 ± 0.03	0.96	0.76 ± 0.02	0.72 ± 0.02	< 0.01
3y-AUROC	0.74 ± 0.02	0.72 ± 0.02	0.04	0.67 ± 0.01	0.67 ± 0.01	0.97
5y-AUROC	0.72 ± 0.02	0.68 ± 0.02	< 0.01	0.66 ± 0.01	0.66 ± 0.01	0.93
Multi-task learning						
* c*-index	0.72 ± 0.01	0.73 ± 0.01	< 0.01	0.68 ± 0.01	0.68 ± 0.01	0.16
2y-AUROC	0.74 ± 0.02	0.74 ± 0.02	0.99	0.75 ± 0.02	0.79 ± 0.03	< 0.01
3y-AUROC	0.77 ± 0.01	0.77 ± 0.01	0.20	0.68 ± 0.01	0.71 ± 0.02	< 0.01
5y-AUROC	0.74 ± 0.01	0.75 ± 0.02	0.07	0.69 ± 0.01	0.71 ± 0.01	< 0.01

The variables between groups were compared using the Student *t*-test

*AUROC* area under the receiver operator curve

Among the multitask learning models, the bone + muscle model showed superior performance in the development set in terms of the *c*-index (0.73 vs. 0.72, *p* < 0.01). In the external test set, the bone + muscle model demonstrated significantly better performance for the 2-year, 3-year, and 5-year AUROCs (2y-AUROC: 0.79 vs. 0.75, *p * < 0.01; 3y-AUROC: 0.71 vs. 0.68, *p* < 0.01; 5y-AUROC: 0.71 vs. 0.69, *p* < 0.01).

### Comparison of imaging-only and clinical multitask model performance for vertebral fracture prediction

**Fig. 3:**
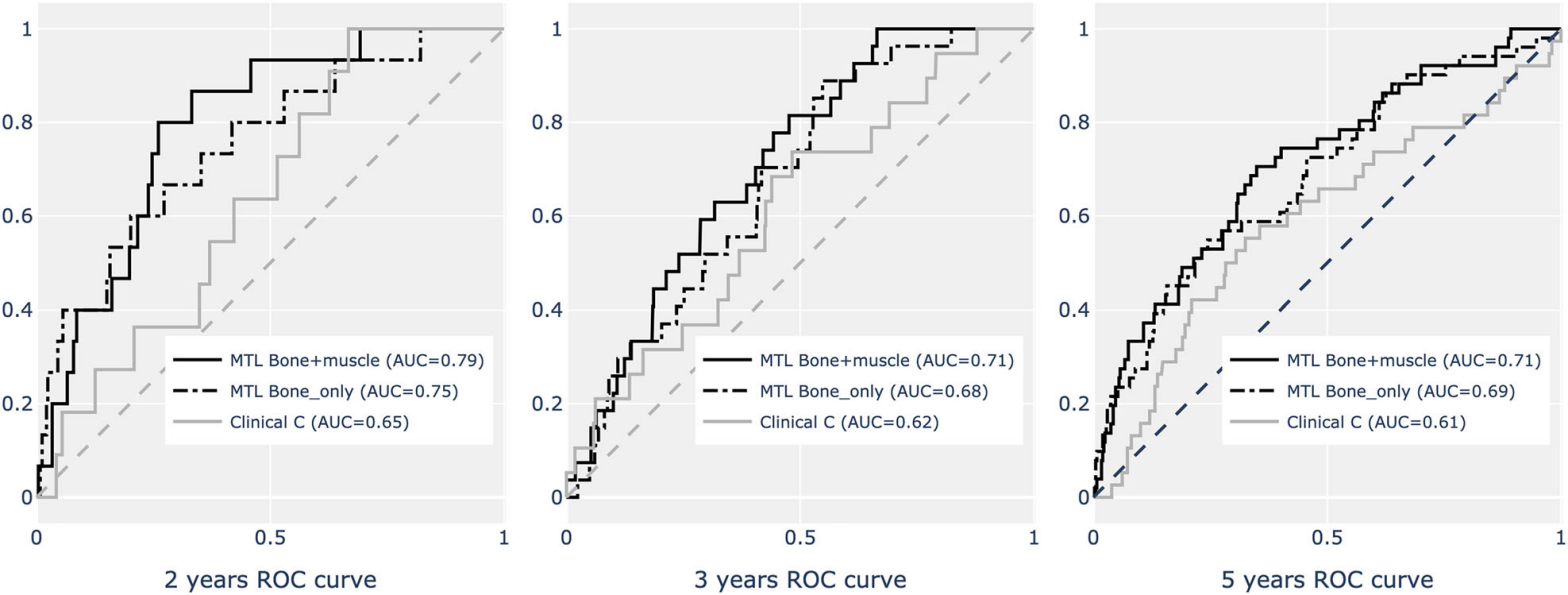
Receiver operating characteristic curves comparing multi-task machine learning and clinical models for vertebral fracture prediction. MTL, multi-task learning; STL, single-task learning

Image-only multitask models significantly outperformed clinical models (including FRAX and various clinical parameters) in both the development and external test sets (Supplementary Table 3 and Fig. 3). In the development set, the image-only multitask model showed a c-index of 0.73 ± 0.01, significantly higher than any clinical model (model A: 0.62 ± 0.01, model B: 0.61 ± 0.01, model C: 0.58 ± 0.01, all *p* < 0.01). In the external test set, the image-only model demonstrated a c-index of 0.68 ± 0.01 compared to the highest-performing clinical model C at 0.61 ± 0.10 (*p* < 0.01).

### Multitask model performance of a combination of image and clinical models

Combining image models with clinical models did not consistently improve the performance over image-only models (Supplementary Table 4). In the development set, the combination of the image model with clinical model C improved the c-index to 0.75 ± 0.01 (*p* < 0.01) compared to the image-only model at 0.73 ± 0.01. However, in some instances, the combination models performed worse, particularly in the external test set, where the combination of image + FRAX model yielded a c-index of 0.51 ± 0.01 compared to the image-only model at 0.68 ± 0.01 (*p* < 0.01).

### Model interpretability using Grad-CAM

To further investigate model interpretability, we applied Grad-CAM to visualize the feature representations of the CNN-based VF risk prediction model. The model consistently focused on the vertebral regions across both the development and external test sets, suggesting that it relied on clinically meaningful areas in a consistent and reliable manner (Supplementary Fig. 2). These findings support the clinical plausibility of the model’s decision-making process.

## Discussion

This study demonstrated the superior performance of CT image-based models over traditional clinical models in predicting VFs. Notably, multitask learning enhanced predictive accuracy when comparing the bone-only image model with the bone-plus-muscle image model. Specifically, the bone and muscle image model employing multitask learning outperformed the bone-only image model in terms of the AUROC, sensitivity, and specificity. This finding suggests that integrating muscle information through multitasking plays a critical role in accurately assessing fracture risk.

In this study, the incorporation of muscle mass information into CT image-based models significantly enhanced the precision of VF risk prediction compared to models using only vertebral bone images. The superior performance of the combined bone and muscle image model aligns with growing evidence suggesting that osteosarcopenia, which is the co-occurrence of bone loss and sarcopenia, is a critical risk factor for osteoporotic fractures [12, 13]. Paravertebral muscles, which play a crucial role in maintaining vertebral stability, have been shown to correlate with VFs through metrics such as cross-sectional area, volume, and fat infiltration [14, 15]. Previous studies have demonstrated that patients with VFs tend to have lower cross-sectional areas and greater fat infiltration in these muscles, supporting the notion that muscle quality and bone density are vital for assessing fracture risk [15, 16]. The presence of myosteatosis or fat infiltration in muscles has also been linked to an increased risk of fractures [17–19]. Therefore, our findings suggest that including paravertebral muscle data in fracture prediction models enhances accuracy by providing a more comprehensive assessment of both bone and muscle health.

Furthermore, multitask learning, simultaneously training VF risk prediction and fracture detection, significantly improved the performance of both tasks. Previous research has shown that learning-related tasks performed together while sharing features enhance performance [20, 21] because they leverage useful domain information from multiple tasks and improve model generalization [20, 22]. Consistent with these findings, we found that although multitask learning increased the computational load and complexity, the knowledge gained from the fracture detection task enhanced the model’s ability to predict VF risk.

The image-based model incorporating both vertebral bone and muscle data outperformed traditional clinical models, confirming previous research on the predictive power of CT-derived bone and muscle information in fracture risk assessment. Unlike earlier studies that focused mainly on bone metrics [23, 24], this study emphasized the importance of integrating muscle information to enhance model performance, reflecting the complex nature of bone strength influenced by both bone density and surrounding muscle architecture. This holistic approach to CT image analysis provides richer data for understanding bone- and muscle-related metabolic diseases and offers insights into fracture risk beyond traditional BMD assessments. This study compared the FRAX with the current model because it is currently the most widely accepted fracture risk assessment tool [25]. Unlike FRAX, which uses clinical information and BMD data, our model relies on the imaging features obtained from abdominal CT scans. The fact that our model demonstrated comparable or superior performance to FRAX suggests that CT images offer more detailed fracture risk information and that CT image-based models could complement traditional methods, especially in settings where opportunistic imaging is already performed.

We found that adding clinical variables to the image-only model did not significantly improve performance, likely because the dataset was matched for age, sex, and BMI; however, some clinical information, such as age and sex, was already partially captured in the images [26, 27]. For example, sex differences in vertebral body and paravertebral muscle sizes, along with a positive correlation between body mass index and these structures, are reflected in imaging [26]. Additionally, bones display age- and sex-related characteristics such as calcification and size changes, which influence model predictions [28]. The increased calcification of vertebral endplates with age suggests that age-related information is inherently present in these images [29]. Lifestyle factors, such as smoking and alcohol consumption, associated with lower muscle mass might also be indirectly captured in muscle imaging, explaining why including these clinical variables does not markedly enhance accuracy [30]. Clinically, this means that future opportunistic CT scans can automatically assess the osteoporotic fracture risk without additional clinical data, thereby streamlining the predictive process. Although the model showed decreased performance in the external test set, the difference was not statistically significant, suggesting that it had a certain degree of generalizability.

This study has several strengths. First, it advances the traditional analysis of BMD by incorporating muscle data into predictive models, which significantly enhances the accuracy of fracture risk prediction. Second, the application of multitask learning techniques further strengthens model performance, enabling more effective utilization of available data and improving both fracture detection and prediction outcomes. Third, the study’s external test, using independent datasets from different hospitals, bolsters the generalizability and robustness of the findings. Additionally, the use of a large dataset with a substantial number of patients provides a solid statistical foundation for the performance of the model and ensures the reliability of the results. Finally, a direct comparison between image-based and traditional clinical models offers clear evidence of the added value that advanced imaging techniques bring to clinical practice, particularly for enhancing fracture risk assessment.

Despite its strengths, this study had several limitations that must be acknowledged. This retrospective design may have introduced biases related to data collection and patient selection, potentially affecting the generalizability of the findings. Furthermore, the primary dataset used for the model development originated from a single institution, which may limit the applicability of the model across different populations and healthcare settings. Another limitation is the study’s focus on fracture prediction within a specific time frame, without assessing long-term outcomes or the model’s performance over extended periods. This temporal limitation restricts the understanding of the model’s effectiveness in predicting fractures beyond the follow-up duration. Additionally, the exclusion of certain patient groups, such as those with poor image quality or previous spinal surgery, further narrowed the scope of the study and may have affected the applicability of the model to a broader patient population.

In conclusion, incorporating muscle features into predictive models significantly enhances the accuracy of VF risk prediction, and the application of multitask learning further improves performance. By simultaneously training the model to detect fractures and predict future fracture risk, multitask learning leverages the shared information between tasks, leading to superior results. From a translational perspective, our model could be integrated into existing radiology workflows by embedding the risk prediction pipeline within picture archiving and communication systems (PACS). This would enable opportunistic VF risk assessment directly from routine abdominal CT scans, without requiring additional imaging or clinical input, thereby facilitating early identification and intervention for high-risk patients.

## Supplementary information

Supplementary information

## Abbreviations

AUROC: Area under the receiver operating characteristic curve
BMD: Bone mineral density
BMI: Body mass index
CT: Computed tomography
FRAX: Fracture risk assessment tool
MOF: Major osteoporotic fracture
STL: Single-task learning
VF: Vertebral fracture
