# Supplementary material for: Enhancing vertebral fracture prediction using multitask deep learning computed tomography imaging of bone and muscle
Source: Eur Radiol. 2025 Dec 1;36(5):4177–86. doi: 10.1007/s00330-025-12049-3 (PMC13086793; doi:10.1007/s00330-025-12049-3)

Supplementary Materials and Methods

Primary Outcome

Morphometric vertebral fractures were confirmed using X-ray or reconstructed CT images by measuring the anterior (H_a), middle (H_m), and posterior (H_p) heights of each vertebral body from T11 to L4. Individuals with no vertebral fractures were classified through a gross visual inspection of vertebral height and shape, ensuring they were within the normal range. The mean and standard deviation (SD) of the ratios of normal vertebral heights were derived from patients without incident fractures. Specifically, the anterior-to-posterior (Ha/Hp), middle-to-posterior (H_m/H_p), and posterior-to-adjacent posterior ($H_{pi}/H_{pi}+1$ and $H_{pi}/H_{pi}-1$) ratios were calculated. A vertebral fracture was defined if any of these ratios were more than 3 SDs below the normal mean for that vertebral level, which was 0.91±0.08, as described in previous studies [1,2].

Sociodemographic factors, including age, sex, and medical history, were obtained from a review of electronic medical records at baseline. Height and body weight were measured by trained staff using standard methods, with a scale and wall-mounted stadiometer, while participants wore lightweight clothing. BMI was calculated as weight divided by height in meters squared (kg/m^2). Current smokers were defined as patients who were smoking during the study period, and current alcohol consumers were those who consumed three or more units of alcohol daily. The use of glucocorticoids was defined as the use of oral glucocorticoids for more than three months at a prednisolone dose of >5 mg or its equivalent. Secondary osteoporosis was defined as osteoporosis resulting from factors other than primary menopause or age-related causes. This includes patients with osteoporosis who also had a concurrent diagnosis of type 1 diabetes, osteogenesis imperfecta in adults, hyperthyroidism, hypogonadism, premature menopause (age <45 years), chronic

malnutrition, malabsorption, or chronic liver disease. [3].

Measurements of BMD and calculations of FRAX®

Bone mineral density (BMD, in grams/cm^2) at skeletal sites (lumbar spine, femoral neck, and total hip) and muscle mass were measured using dual-energy X-ray absorptiometry (DXA). The Discovery W system (Hologic, Inc.) was used in the discovery set, while the GE Prodigy system (GE Healthcare) was used in the external validation set. Measurements were analyzed according to the manufacturer's guidelines at baseline. The precision error for BMD (% coefficient of variation, CV) was 1.7% for the lumbar spine, 1.8% for the femoral neck, and 1.7% for the total hip. For lumbar spine BMD, the L1–L4 region was typically used for analysis. However, if L1–L4 was unsuitable due to a compression fracture or severe sclerotic changes, the L2–L4 region was used instead. The instruments were calibrated using anthropomorphic phantoms.

The 10-year absolute risks of hip and osteoporotic fractures were calculated using the World Health Organization's FRAX® tool, specifically the Korea-specific version available online from the University of Sheffield (https://www.sheffield.ac.uk/FRAX/tool.aspx?country=25). The FRAX algorithm incorporates the following parameters: femoral neck BMD T-score, age, sex, body mass index, previous history of fracture, parental history of hip fracture, secondary osteoporosis, current smoking status, recent use of corticosteroids, presence of rheumatoid arthritis, and consumption of three or more alcoholic beverages per day.

CT protocols

All patients underwent contrast-enhanced abdominal CT using 64- to 256-multidetector CT

devices (Brilliance 64 or Ingenuity or iCT or IQon, Philips; SOMATOM Force or SOMATOM Definition, Siemens; Discovery CT750 or Revolution, GE Medical Systems). The tube voltage, gantry rotation time, pitch, matrix, field of view, and slice thickness were 80–120 kVp, 0.5 seconds, 0.6, 512 × 512, 271 × 271 – 499 × 499, and 2.5–5 mm, respectively. We applied the technology available from each vendor for automatic tube current modulation. Images were reconstructed using semi-smooth quantitative body kernels. We used portal phase images, which were obtained 70 s after administering iodinated contrast medium (350 mg/mL) into the peripheral vein of the upper extremity via an automatic power injector at a total dosage of 1.5 mL/kg over 30 s.

Image preprocessing

Standard medical image preprocessing steps were applied as an initial step. Each CT scan was resampled to a uniform voxel spacing, followed by intensity clipping to the range of (0, 1000) Hounsfield units. The clipped images were then normalized using min–max normalization.

Building on these standardized inputs, we designed a data preprocessing pipeline to extract features relevant to vertebral fracture risk from 3D CT scans. A segmentation model, trained on a labeled CT dataset with manually annotated vertebrae and muscle regions, was used to generate masks for the vertebral bodies (T12-L4) and surrounding muscles, specifically the left and right autochthon and iliopsoas muscles. Each 3D CT scan was divided into five 3D patches of size 24×128×224, each containing a vertebral body and its associated muscle masks. Only the CT image data corresponding to the masked regions, namely the vertebral bodies and the left and right autochthon and iliopsoas muscles, were retained by overlaying

the masks onto the CT scans. For the fracture detection dataset, vertebrae with fractures were excluded, ensuring that the model relied solely on non-fractured vertebrae for fracture prediction. To handle variations in the number of vertebrae per scan, three non-fractured vertebrae were randomly sampled from the remaining set. For each selected 3D patch, nine axial 2D CT patches were extracted. The number of vertebrae sampled, the dimensionality of the data (2D or 3D), and the 2D patch sampling strategy were determined empirically to optimize model performance.

Implementation

We employed a ConvNeXT Tiny model, which has approximately 28.6 million parameters, pre-trained on ImageNet-1K as the feature extractor for multitask learning [(4)]. We selected ConvNeXT for its strong balance between accuracy, scalability, and efficiency, while retaining the simplicity and robustness of ConvNets.

The segmentation models used in the image preprocessing pipeline were TotalSegmentator and a modified 2D U-Net, which were applied to extract vertebrae and muscle regions [(5,6)]. Specifically, TotalSegmentator was first used to segment vertebrae T12 to L4 as well as the surrounding muscle regions. To further isolate the vertebral body portion, we applied a modified 2D U-Net trained on a manually labeled 3D CT dataset, which refined the vertebrae segmentation by excluding posterior elements and retaining only the vertebral bodies.

The classification head consisted of two randomly initialized multilayer perceptrons, while the hazard head utilized a survival analysis model architecture [(7)]. To balance the contributions of each task during training in the multitask learning framework, we applied the Nash-MTL technique. This approach enabled effective optimization of both classification and

hazard prediction objectives [8].

For model evaluation, we randomly selected 20% of the development dataset as a validation set and used the remaining 80% for training. Data augmentation techniques included random affine transformations with scaling factors ranging from 0.8 to 1.2, rotations between -5° and 5°, and random left-right flipping with a probability of 0.5. The model was trained for 60 epochs with a batch size of 64 using the AdamW optimizer with an initial learning rate of 1×10^{-4}. During training, the model weights that achieved the highest concordance index (c-index) on the development set were saved and used for evaluation. The OneCycleLR scheduler was used to adjust the learning rate during training. For the binary classification task, cosine loss was employed. All components were implemented using the PyTorch library in Python.

Statistical analyses

The variables between groups were compared using the Student's t-test. For the fracture detection task, the performance of the deep learning model was evaluated using the area under the receiver operating characteristic curve (AUROC), accuracy, sensitivity, and specificity. For the vertebral fracture risk prediction task, model performance was evaluated using the c-index, 2-year AUROC (2y-AUROC), 3-year AUROC (3y-AUROC), and 5-year AUROC (5y-AUROC). The same code was run 10 times with different random seeds to evaluate performance for both tasks. Youden’s J statistic was used to choose the best operating point.

The image-only model predicts future fracture risk by passing preprocessed CT scans through a CNN-based feature extractor followed by a hazard head. In contrast, clinical

models predict future fracture risk by passing several clinical features through a lightweight feature extractor, consisting of two multilayer perceptrons, followed by a hazard head. Based on the input clinical features, the clinical models are divided into three types: Model A uses two variables—age and sex; Model B adds BMI to these; and Model C further includes current smoker, current drinker, use of steroids, and secondary osteoporosis. Lastly, in the combination of the image and clinical model, features from the CNN-based feature extractor and the lightweight feature extractor are concatenated before being passed through the hazard head[(9)]. Statistical analyses were performed using the PyTorch, Scikit-learn, and Lifelines libraries in Python.

References

1. Genant HK, Wu CY, van Kuijk C, Nevitt MC. Vertebral fracture assessment using a semiquantitative technique. J Bone Miner Res. Sep 1993;8(9):1137-48. Epub 1993/09/01.
2. Shin CS, Kim MJ, Shim SM, Kim JT, Yu SH, Koo BK, et al. The prevalence and risk factors of vertebral fractures in Korea. J Bone Miner Metab. Mar 2012;30(2):183-92. Epub 2011/07/21.
3. Kanis JA, Johansson H, McCloskey EV, Liu E, Åkesson KE, Anderson FA, et al. Previous fracture and subsequent fracture risk: a meta-analysis to update FRAX. Osteoporos Int. Dec 2023;34(12):2027-45. Epub 2023/08/11.
4. Liu Z, Mao H, Wu C-Y, Feichtenhofer C, Darrell T, Xie S. A convnet for the 2020s. Proceedings of the IEEE/CVF conference on computer vision and pattern recognition2022. p. 11976-86.
5. Wasserthal J, Breit HC, Meyer MT, Pradella M, Hinck D, Sauter AW, et al. TotalSegmentator: robust segmentation of 104 anatomic structures in CT images. Radiol Artif Intell 2023;5:e230024.
6. Isensee F, Kickingereder P, Wick W, Bendszus M, Maier-Hein KH. Brain tumor segmentation and radiomics survival prediction: contribution to the BRATS 2017 challenge. In: Crimi A, Bakas S, editors. Brainlesion: glioma, multiple sclerosis, stroke and traumatic brain injuries. BrainLes 2017. Lecture Notes in Computer Science. Cham: Springer; 2018. p. 287–97.
7. Mikhael PG, Wohlwend J, Yala A, Karstens L, Xiang J, Takigami AK, et al. Sybil: a validated deep learning model to predict future lung cancer risk from a single low-dose chest computed tomography. Journal of Clinical Oncology. 2023;41(12):2191-

200.

8. Navon A, Shamsian A, Achituve I, Maron H, Kawaguchi K, Chechik G, et al. Multi-task learning as a bargaining game. arXiv preprint arXiv:220201017. 2022.

9. Huang SC, Pareek A, Seyyedi S, Banerjee I, Lungren MP. Fusion of medical imaging and electronic health records using deep learning: a systematic review and implementation guidelines. NPJ Digit Med. 2020;3(1):136.

Supplement Table 1. Clinical characteristics according to baseline fracture status for classification task

	Development set				External test set				
	**Baseline fracture (-)**	**Baseline fracture (+)**	**Total**	p^a	**Baseline fracture (-)**	**Baseline fracture (+)**	**Total**	p^a	p^b
**Number of patients**	1144	1409	2553		746	760	1506		
**Age**	77.5 ± 10.0	77.3 ± 8.8	77.4 ± 9.6	0.06	74.0 ± 9.7	74.6 ± 8.5	74.3 ± 9.2	0.08	<0.01
**Female**	776 (67.9%)	950 (67.4%)	1726 (67.6%)	0.12	498 (66.8%)	545 (71.7%)	1043 (69.3%)	0.07	0.16
**Height, cm**	157.6 ± 8.9	156.9 ± 8.8	157.2 ± 8.8	0.08	157.9 ± 8.1	156.1 ± 8.7	157.0 ± 8.4	< 0.01	0.48
**Weight, kg**	57.5 ± 10.7	54.8 ± 11.0	56.0 ± 10.9	< 0.01	59.4 ± 10.2	57.4 ± 10.5	58.4 ± 10.4	< 0.01	< 0.01
**BMI, kg/m^2**	23.7 ± 3.4	23.4 ± 3.7	23.5 ± 3.6	0.26	23.8 ± 3.4	23.5 ± 3.7	23.6 ± 3.6	0.22	< 0.01
**Current smoker**	239 (20.9%)	321 (22.8%)	560 (21.9%)	0.27	25 (3.7%)	27 (3.7%)	52 (3.7%)	>0.99	< 0.01
**Current drinker**	227 (19.8%)	257 (18.2%)	484 (19.0%)	0.33	57 (8.3%)	34 (4.7%)	91 (6.4%)	0.01	< 0.01
**Use of steroids**	276 (24.1%)	420 (29.8%)	696 (27.3%)	0.01	9 (1.2%)	23 (3.0%)	32 (2.1%)	0.02	< 0.01
**Secondary osteoporosis**	92 (4.6%)	141 (12.3%)	233 (9.1%)	< 0.01	61 (8.1%)	82 (10.7%)	143 (9.5%)	0.06	< 0.01
**Lumbar spine BMD**	0.913 ± 0.198	0.847 ± 0.172	0.864 ± 0.181	< 0.01	0.949 ± 0.204	0.872 ± 0.180	0.899 ± 0.193	< 0.01	< 0.01
**Femur neck BMD**	0.672 ± 0.159	0.589 ± 0.134	0.613 ± 0.147	< 0.01	0.747 ± 0.145	0.686 ± 0.133	0.708 ± 0.140	< 0.01	< 0.01
**Total hip BMD**	0.766 ± 0.154	0.668 ± 0.139	0.694 ± 0.150	< 0.01	0.791 ± 0.151	0.722 ± 0.142	0.746 ± 0.149	< 0.01	< 0.01

BMI, body mass index; BMD, bone mineral density. Use of steroids was defined as the use of prednisolone 5 mg daily or equivalent over 3 months. Numbers are presented as numbers (percentages) or mean (standard deviation). The variables between groups were compared using the Student t-test for continuous variables and the $\chi 2$ test for categorical variables. p^a represents p value between groups with and without baseline fractures. p^b represents p value between development and external test sets.

Supplement Table 2. Comparisons of performances of image models in detecting baseline fracture

	Development set			*External test set*		
	**Bone-only**	**Bone + Muscle**	***P***	**Bone-only**	**Bone + Muscle**	***P***
**AUROC**	0.81 ± 0.01	0.82 ± 0.01	0.01	0.76 ± 0.01	0.80 ± 0.01	<0.01
**Accuracy**	0.75 ± 0.01	0.73 ± 0.01	<0.01	0.7 ± 0.01	0.72 ± 0.01	<0.01
**Sensitivity**	0.82 ± 0.01	0.64 ± 0.03	<0.01	0.68 ± 0.01	0.6 ± 0.01	<0.01
**Specificity**	0.66 ± 0.01	0.83 ± 0.01	<0.01	0.71 ± 0.01	0.84 ± 0.01	<0.01

AUROC, the area under the receiver operator curve. The variables between groups were compared using the Student t-test.

Supplement Table 3. Comparisons of performances of image and clinical models in predicting vertebral fractures

	c-index	*p*	2y-AUROC	*p*	3y-AUROC	*p*	5y-AUROC	*p*
**Development set**								
**Image-only**	0.73 ± 0.01	Reference	0.74 ± 0.03	Reference	0.77 ± 0.02	Reference	0.75 ± 0.02	Reference
**FRAX, MOF**	0.56 ± 0.01	<0.01						
**FRAX, hip**	0.56 ± 0.01	<0.01						
**Clinical model A**	0.62 ± 0.01	<0.01	0.65 ± 0.02	<0.01	0.63 ± 0.01	<0.01	0.69 ± 0.01	<0.01
**Clinical model B**	0.61 ± 0.01	<0.01	0.62 ± 0.04	<0.01	0.63 ± 0.03	<0.01	0.66 ± 0.02	<0.01
**Clinical model C**	0.58 ± 0.01	<0.01	0.61 ± 0.05	<0.01	0.59 ± 0.04	<0.01	0.62 ± 0.03	<0.01
**External test set**								
**Image-only**	0.68 ± 0.01	Reference	0.79 ± 0.02	Reference	0.71 ± 0.01	Reference	0.71 ± 0.01	Reference
**FRAX, MOF**	0.66 ± 0.01	<0.01						
**FRAX, hip**	0.56 ± 0.01	<0.01						
**Clinical model A**	0.56 ± 0.01	<0.01	0.60 ± 0.04	<0.01	0.55 ± 0.03	<0.01	0.58 ± 0.01	<0.01

**Clinical model B**	0.53 ± 0.04	<0.01	0.59 ± 0.04	<0.01	0.56 ± 0.03	<0.01	0.56 ± 0.04	<0.01
**Clinical model C**	0.60 ± 0.07	<0.01	0.65 ± 0.04	<0.01	0.62 ± 0.05	<0.01	0.61 ± 0.10	0.05

Numbers are presented as mean ± standard deviation. AUROC, the area under the receiver operator curve; MOF, major osteoporotic fracture. Image model represents the model using bone and muscle. Model A includes age and sex, model B additionally includes body mass index, and model C additionally includes history of drinking, smoking, and possible secondary osteoporosis.

Supplement Table 4. Comparisons of performances of image and combination of image and clinical models

	c-index	*p*	2y-AUROC	*p*	3y-AUROC	*p*	5y-AUROC	*p*
**Development set**								
**Image-only**	0.73 ± 0.01	Reference	0.74 ± 0.03	Reference	0.77 ± 0.02	Reference	0.77 ± 0.02	Reference
**Image + FRAX (MOF)**	0.73 ± 0.01	0.57	0.71 ± 0.03	0.02	0.72 ± 0.01	<0.01	0.76 ± 0.01	0.28
**Image + FRAX (hip)**	0.69 ± 0.01	<0.01	0.71 ± 0.02	0.02	0.72 ± 0.01	<0.01	0.71 ± 0.01	<0.01
**Image + clinical model A**	0.72 ± 0.01	0.02	0.77 ± 0.03	0.06	0.77 ± 0.03	1.00	0.72 ± 0.01	<0.01
**Image + clinical model B**	0.73 ± 0.01	0.15	0.73 ± 0.02	0.47	0.75 ± 0.01	<0.01	0.72 ± 0.01	<0.01
**Image + clinical model C**	0.75 ± 0.01	<0.01	0.78 ± 0.04	<0.01	0.79 ± 0.01	0.05	0.76 ± 0.01	0.12
**External test set**								
**Image-only**	0.68 ± 0.01	Reference	0.76 ± 0.02	Reference	0.69 ± 0.01	Reference	0.71 ± 0.01	Reference
**Image + FRAX (MOF)**	0.51 ± 0.01	<0.01	0.54 ± 0.02	<0.01	0.50 ± 0.02	<0.01	0.56 ± 0.01	0.02
**Image + FRAX (hip)**	0.49 ± 0.01	<0.01	0.29 ± 0.03	<0.01	0.34 ± 0.04	<0.01	0.50 ± 0.50	<0.01
**Image + clinical model A**	0.62 ± 0.01	<0.01	0.74 ± 0.02	0.71	0.65 ± 0.02	<0.01	0.63 ± 0.01	<0.01

**Image + clinical model B**	0.65 ± 0.01	<0.01	0.73 ± 0.04	0.44	0.67 ± 0.02	<0.01	0.66 ± 0.01	<0.01
**Image + clinical model C**	0.64 ± 0.02	<0.01	0.81 ± 0.02	<0.01	0.63 ± 0.04	<0.01	0.66 ± 0.02	<0.01

Numbers are presented as mean ± standard deviation. AUROC, the area under the receiver operator curve; MOF, major osteoporotic fracture. Image model represents the model using bone and muscle. Model A includes age and sex, model B additionally includes body mass index, and model C additionally includes history of drinking, smoking, and possible secondary osteoporosis.

Supplement Figure 1. Flowchart of Participant Selection for Study on Vertebral Fracture (A) Classification and (B) Prediction

CT, computed tomography; BMI, body mass index.

(A)

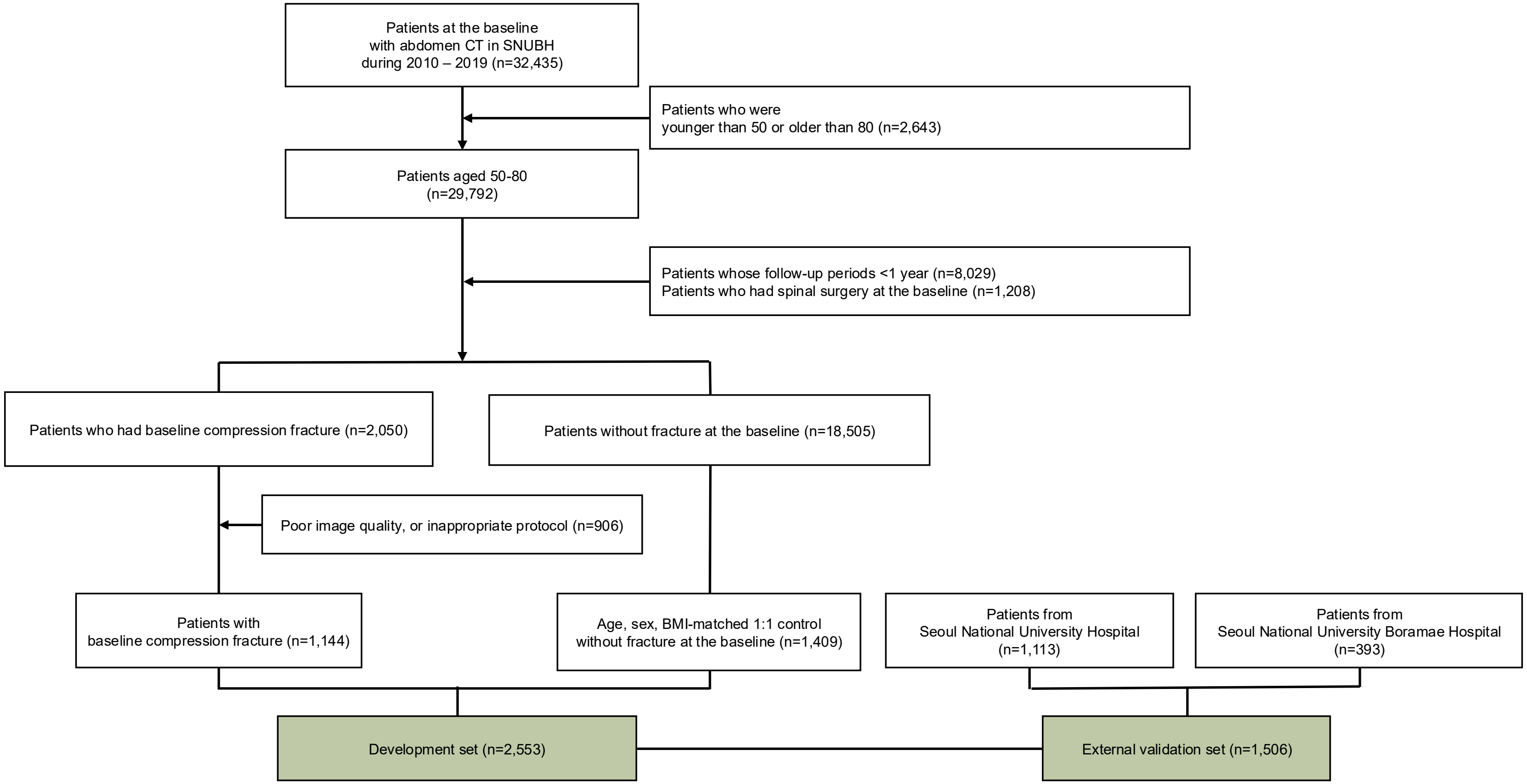

(B)

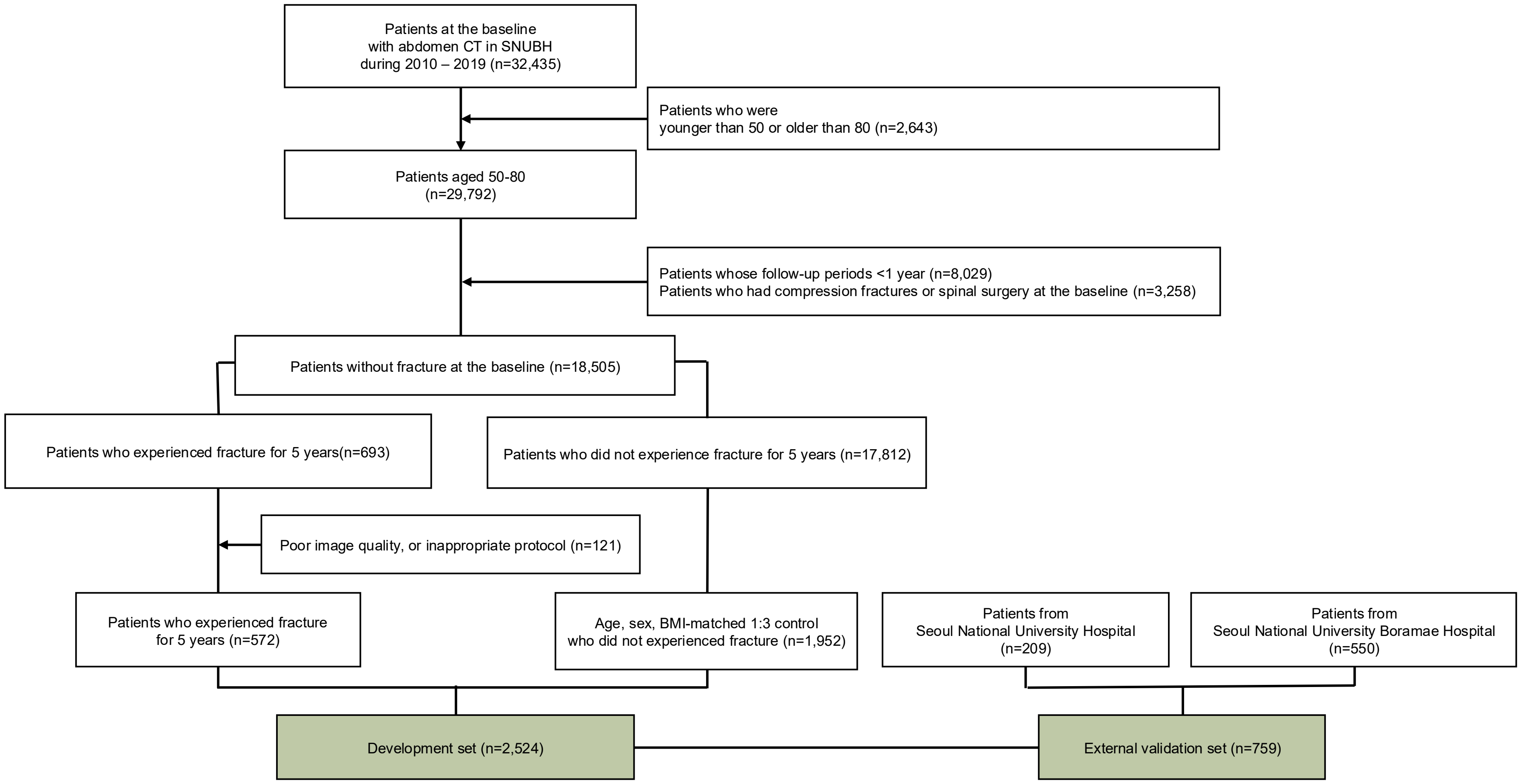
Patients at the baseline
with abdomen CT in SNUBH
during 2010 – 2019 (n=32,435)
Patients who were
younger than 50 or older than 80 (n=2,643)
Patients aged 50-80
(n=29,792)
Patients whose follow-up periods <1 year (n=8,029)
Patients who had compression fractures or spinal surgery at the baseline (n=3,258)
Patients without fracture at the baseline (n=18,505)
Patients who experienced fracture for 5 years(n=693)
Patients who did not experience fracture for 5 years (n=17,812)
Poor image quality, or inappropriate protocol (n=121)
Patients who experienced fracture
for 5 years (n=572)
Age, sex, BMI-matched 1:3 control
who did not experienced fracture (n=1,952)
Patients from
Seoul National University Hospital
(n=209)
Patients from
Seoul National University Boramae Hospital
(n=550)
Development set (n=2,524)
External validation set (n=759)

Supplement Figure 2. Grad-CAM Visualizations for Model Interpretability on (A) the Development Set and (B) the External Test Set

(A) Development set **(B) External test set**

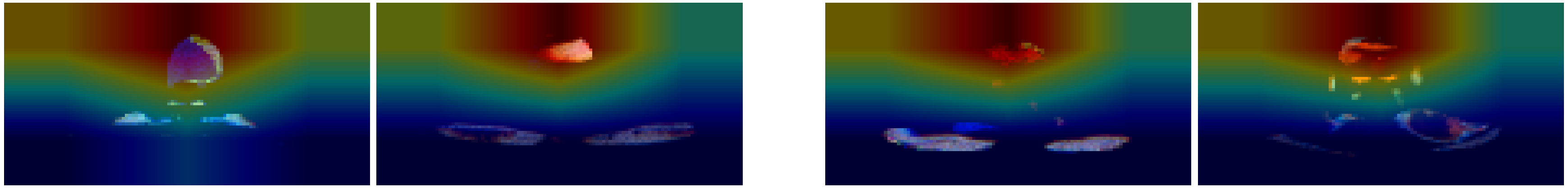

Supplement: Supplementary file 1 — Supplementary information [file 330_2025_12049_MOESM1_ESM.pdf]
